# Magnesium ions regulate mesenchymal stem cells population and osteogenic differentiation: A fuzzy agent-based modeling approach

**DOI:** 10.1016/j.csbj.2021.07.005

**Published:** 2021-07-09

**Authors:** Jalil Nourisa, Berit Zeller-Plumhoff, Heike Helmholz, Bérengère Luthringer-Feyerabend, Vladimir Ivannikov, Regine Willumeit-Römer

**Affiliations:** Helmholtz Zentrum Hereon, Institute of Metallic Biomaterials, Max-Planck-Straße 1, 21502 Geesthacht, Germany

**Keywords:** Magnesium ions, Agent-based simulation, Fuzzy logic-based approach, Mesenchymal stem cells, Cell population, Osteogenic differentiation, Approximate Bayesian calculation

## Abstract

Mesenchymal stem cells (MSCs) are proliferative and multipotent cells that play a key role in the bone regeneration process. Empirical data have repeatedly shown the bioregulatory importance of magnesium (Mg) ions in MSC growth and osteogenesis. In this study, we propose an agent-based model to predict the spatiotemporal dynamics of the MSC population and osteogenic differentiation in response to Mg^2+^ ions. A fuzzy-logic controller was designed to govern the decision-making process of cells by predicting four cellular processes of proliferation, differentiation, migration, and mortality in response to several important bioregulatory factors such as Mg^2+^ ions, pH, BMP2, and TGF-β1. The model was calibrated using the empirical data obtained from three sets of cell culture experiments. The model successfully reproduced the empirical observations regarding live cell count, viability, DNA content, and the differentiation-related markers of alkaline phosphate (ALP) and osteocalcin (OC). The simulation results, in agreement with the empirical data, showed that Mg^2+^ ions within 3–6 mM concentration have the highest stimulation effect on cell population growth. The model also correctly reproduced the stimulatory effect of Mg^2+^ ions on ALP and its inhibitory effect on OC as the early and late differentiation markers, respectively. Besides, the numerical simulation shed light on the innate cellular differences of the cells cultured in different experiments in terms of the proliferative capacity as well as sensitivity to Mg^2+^ ions. The proposed model can be adopted in the study of the osteogenesis around Mg-based implants where ions released due to degradation interact with local cells and regulate bone regeneration.

## Graphical abstract

**Figure:**
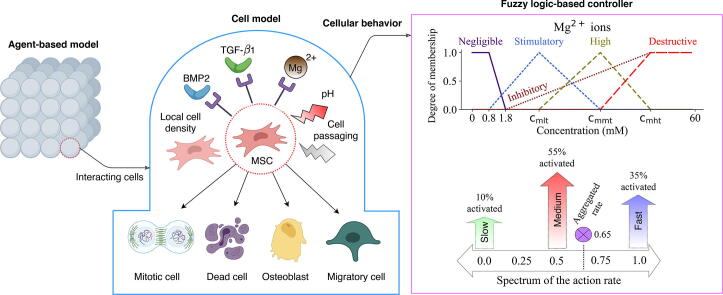

## 1 Introduction

Mesenchymal stem cells (MSCs) are the key players in bone fracture healing [1]. MSCs increase cell population through a fast proliferation process and differentiate into multiple cell types involved in bone tissue regeneration, in particular osteoblasts [2]. The proliferation process occurs through a cascade of cell cycle events including the two major processes of DNA synthesis and actual division of the parent cell into two daughter cells [2]. The specialization of MSCs toward osteoblasts involves a complex intracellular interaction and is shown to occur continuously with recognizable intermediate cells such as osteoprogenitors and pre-osteoblasts [3]. During osteogenic differentiation, MSCs experience a decline in proliferative capacity and gain osteoblastic properties [4]. The onset of MSC differentiation to osteoblasts and the progression along this lineage are controlled by various signals such as growth factors, mechanical signals, and biomaterials [5], [6].

Magnesium (Mg)-based biomaterials are biodegradable which makes them an attractive choice in the orthopedic application and medical-technical industry [6], [7]. Mg implants degrade at the implantation site resulting in an alteration in the microenvironment of the local tissue. Mg^2+^ ions released during degradation are demonstrated by several *in vivo* and *in vitro* studies to regulate gene and protein expressions associated with cell growth and osteogenesis [7], [8], [9]. The release of Mg^2+^ ions in high concentrations is also associated with the alteration of the microenvironment pH [9], causing an alkaline condition and consequently interfering with a broad range of physiological processes [10], [11], [12]. In order to design an effective Mg-based implant, it is essential to study the bioregulatory mechanisms of Mg^2+^ ions and identify the optimal conditions to promote osteogenic activities [13], [14]. So far, the empirical approach has been the only means to study the bioregulatory effect of Mg-based materials. In this study, we aim at numerically investigating the physiological interaction of Mg^2+^ ions with MSCs.

We choose agent-based modeling (“agent-based model” and “agent-based modeling” are both abbreviated as ABM) to address the current problem. ABM provides a multiscale investigation of a system as a direct observation can be made on individual cells while the cumulative results are captured at the population level [19], [20], [21]. ABM has been widely used in the literature to study cellular responses [15], [17], [18]. A common challenge in ABM is the abstraction of cellular behavior which requires an algorithm to correctly govern the decision-making process [22], [23]. Such an algorithm receives cellular inputs at the microscale and predicts cellular behavior. Several approaches have been proposed in the literature to simulate the decision-making process such as simple rule definition, differential equations, logic-based approach, and artificial neural networks [15], [24], [25], [26]. Fuzzy logic (FL)-based have shown great potential in resolving technical barriers between experimental and simulation experts thanks to its plain language [27]. In this approach, knowledge about a system can be formulated in the form of IF-THEN statements, in which IF and THEN are conditions and results, respectively. This plain language can potentially ease the involvement of people with domain knowledge in the rapid development of computer models. Since FLB models can define a system without precise mechanistic information, it is possible to leverage qualitative knowledge in numerical modeling which would be otherwise difficult or impossible using other simulation approaches that require real-valued variables [27]. Due to these advantages, the FL-based approach has already been repeatedly employed in the numerical investigation of bone regeneration [28], [29], [30].

In this study, we propose a fuzzy agent-based model to simulate the spatiotemporal dynamics of mesenchymal stem cell population and osteogenic differentiation in response to Mg^2+^ ions. To this end, the available information in the literature regarding the bioregulatory effect of Mg^2+^ ions in tandem with several other important factors is curated and tailored as fuzzy logic rules. Differential equations are used to describe the dynamics of growth factors. The data obtained from three sets of published cell-culture experiments are used to estimate the model’s parameters by employing approximate Bayesian calculation.

## 2 Materials and methods

**Fig. 1:**
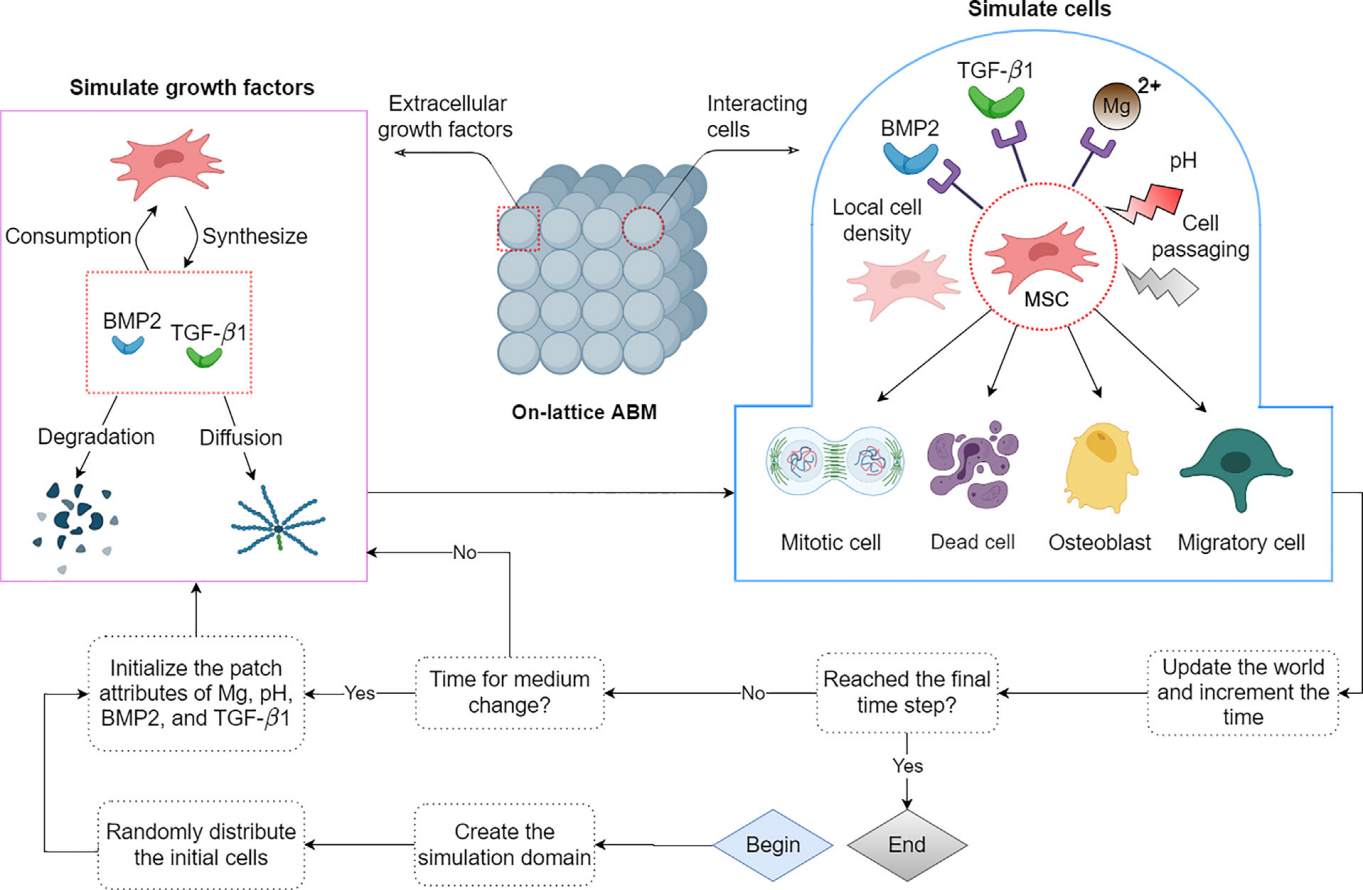
The workflow of the ABM in this study. Once the model is initialized, cells and growth factors are simulated iteratively, and the simulation world is updated according to their results. For culture experiments longer than 3 days, the content of the growth factors and pH value is reset to the initial values every 2.5 days accounting for the process of medium change [31]. Four cellular events of proliferation, migration, osteoblastic differentiation, and mortality are simulated which are affected by multiple environmental factors. The dynamics of the growth factors are driven by cellular production, cellular consumption, degradation, and diffusion. Each iteration in our simulation represents one hour [34], [35]. Some elements of the graph are created with BioRender.com.

The proposed ABM in this study consists of three components; a cell model, a model to simulate growth factors, and a coordinator. As shown in Fig. 1, the coordinator initializes the simulation, iteratively executes cells and growth factors, and updates the simulation world. In the rest of this section, we first give an introduction to the ABM. Then, the construction of the cell model is elaborated in detail. Lastly, the process of sensitivity analysis and the calibration is introduced.

### 2.1 The agent-based model

We use a lattice-based approach where the occupancy of each patch is limited to one cell at a time. A three-dimensional (3D) space with 8 layers in the z-direction is created to account for the observation that the osteoblastic differentiation of MSCs generates more than four cell layers *in vitro* [31]. Further information regarding the geometry of the model, the initialization, and the boundary conditions can be found in section S1 in the supplements. The dynamics of growth factors are simulated similar to Ribeiro et al [32] and are elaborated in section S2 in the supplement. The software used to develop the model can be found in section S3 in the supplement. The source code of the present model can be found online [33].

### 2.2 The cell model

**Fig. 2:**
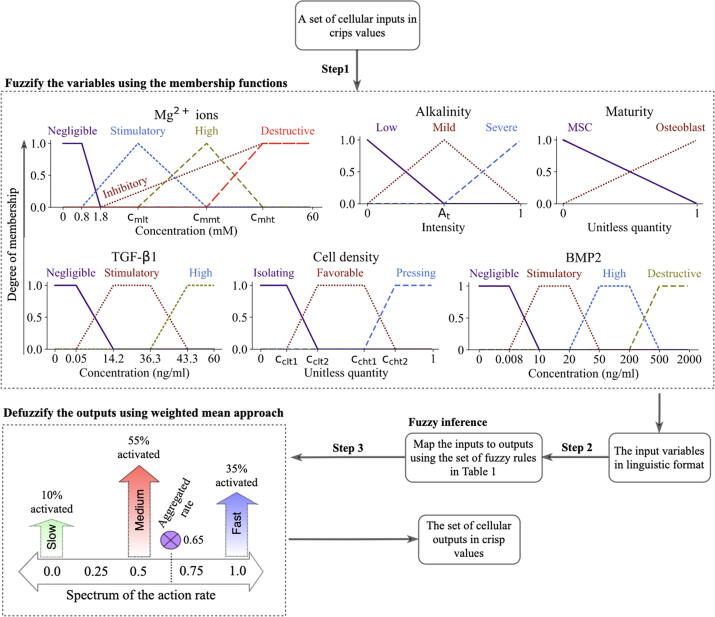
The complete calculation process of the FL controller in this study. The inputs of the FL controller are converted to linguistic variables using the membership functions (step 1). The decision-making center, which comprises the fuzzy rules, receives the fuzzified inputs and determines which fuzzy outputs are activated (step 2). The final output of the controller is calculated by averaging on the activated outputs using the weighted mean approach (step 3).

We define five inputs of Mg^2+^ ions, alkalinity, TGF-β1, BMP2, and cell density as the bioregulatory cues of the cellular behavior. In addition, two intrinsic factors of maturity and DNA damage are simulated to influence cellular functions. The cell model predicts the four cellular behaviors of proliferation, differentiation, mortality, and migration. A Mamdani-type FL controller is implemented to compute the intensity of the cellular actions as a cumulative result of the stimulatory signal. The FL controller operates in three steps of fuzzification, inference, and defuzzification as shown in Fig. 2 [36].

#### 2.2.1 Cellular inputs and fuzzification

**Maturity** represents the degree of maturation of MSCs along the line of osteoblast lineage, similar to Krinner et al [17]. The spectrum of maturation is divided into two phases of the early and late differentiation, marked by a parameter termed maturity threshold (M_t_). Maturity linearly increases by cell commitment to the differentiation process at each time step of the simulation. During this process, cells lose their characteristics as MSCs and obtain osteoblastic characteristics. Maturity is fuzzified using two linguistic terms as shown in Fig. 2.

**DNA damage** stands for the irreversible cellular impairment due to harsh environmental conditions. In our simulations, DNA damage can be caused by either cell passaging due to the usage of chemical products and mechanical forces [37] or exposure to a high pH (pH_t_) [11]. In the simulation, DNA damage can occur by a base chance (γ_C_) at the beginning of the simulation, accounting for cell passaging, or by one hour of exposure (one step of the simulation) to pH_t_ [11]. The factor of DNA damage is simulated as a crisp quantity that takes the value of either 1 (*high*) or 0 (*low*).

**Mg^2+^ ions** are shown to regulate cellular responses depending on the applied concentration, exposure duration, and the state of cell differentiation [9], [38], [39], [40]. Mg^2+^ ions within the concentration range of 2–10 mM enhance cell metabolism and upregulate proliferation and early differentiation rate [38], [40], [41], [42], [43]. However, Mg^2+^ ions above 1.8 mM have shown an inhibitory effect on late differentiation rate and matrix mineralization [42], [13]. Also, Mg^2+^ ions at concentrations ranging from 20 to 40 mM is reported toxic and can reduce cell viability [42], [44], [45]. To account for these observations, we define five linguistic terms to fuzzify the input factor of Mg^2+^ ions (see Fig. 2). The concentration of Mg^2+^ ions below 0.8 mM, which is used in cell culture medium (minimal essential Medium-MEM), are set to *negligible* with no stimulatory effect [9], [43]. The *inhibitory* level takes into account the inhibitory effect of Mg^2+^ ions on the late differentiation process. The *stimulatory* level simulates the stimulatory role of Mg^2+^ ions on proliferation and early differentiation process. The toxic effect of Mg^2+^ ions in high concentrations is modeled by *destructive* level. Three parameters of $c_{\mathrm{mlt}}$, $c_{\mathrm{mmt}}$, and $c_{\mathrm{mht}}$ mark the peak occurrence of stimulatory, high, and destructive levels.

**Alkalinity** is defined as the sudden change of the ambient pH with respect to the intracellular pH. Mg^2+^ ions are reported to alter microenvironmental pH [9] (see Fig. S1-A in the supplements), causing an alkaline condition which disrupts cellular reactions. In contrast to permanent DNA damage, we assume that cells can recover from mild alkalinity [9]. This process happens by the adjustment of the cell’s internal pH with respect to the ambient pH over time with a constant rate (r_r_) [9]. Alkalinity can significantly affect cellular reaction depending on the severity [11]. Several minutes of exposure to severe alkalinity is reported to cause cell contraction and detachment from the culture surface [12]. Severe alkalinity can compromise human MSC renewal capability and growth and thereby downregulate proliferation rate [10]. High alkalinity also reduces cell viability in culture experiments [10]. However, a mild alkaline environment with a pH as high as 8.5 has shown no significant negative effect on osteoblast differentiation [10]. Three linguistic terms are assigned to alkalinity during the fuzzification process as shown in Fig. 2. We assume that both *mild* and *severe* alkalinity can compromise cellular events of proliferation and health, while only *severe* alkalinity affects the differentiation process. The parameter of A_t_ marks the start of *severe* level.

**BMP2** is the most potent BMP heterodimer in the stimulation of osteoblast differentiation [46], [47], [48]. BMP2 is shown to affect cell proliferation in a concentration-dependent fashion. BMP2 at the concentration of 10–20 ng/mL promotes cell proliferation [49], [50]. However, BMP2 has shown no effect and a negative effect within the concentration ranges of 50–200 ng/mL and 500–2000 ng/mL, respectively [51], [52]. BMP2 at the concentration of 10–20 ng/mL has also shown a stimulatory impact on osteogenic differentiation [49], [50]. BMP2 at the concentration of 500–2000 ng/mL stimulates cell apoptosis and thereby decreases cell viability [51]. We assign four membership levels to the input of BMP2 as shown in Fig. 2. The *stimulus* level starts from the concentration of 0.008 ng/mL as the lower bound of the physiological concentration reported in *in vitro* experiments [53], [54], [55].

**TGF-β1** is an important regulatory factor in every stage of bone regeneration [16], [56], [46]. Within the physiological concentration of 14.2–36.3 ng/mL, TGF-β1 is shown to stimulate the proliferation process, promote early osteoblast differentiation, and inhibit the later phase of differentiation [56], [46]. Within the physiological range, TGF-β1 is also shown to block the natural process of apoptosis [57]. The input variable of TGF-β1 is fuzzified according to Fig. 2, where the concentration of 0.05 ng/mL marks the beginning of the *stimulatory* level [58], [59].

**Cell density** is calculated as the normalized number of cells in one patch neighborhood. Cell density is another important factor that is known to affect various cellular reactions such as migration, proliferation, differentiation, and mortality [60]. High cell density results in a phenomenon termed contact inhibition that halts cell growth and initiates the differentiation process [61], [62]. Contact inhibition also affects cell migration as cells intend to move toward an area with less crowdedness to receive better nutrition and oxygen [63]. A high degree of crowdedness is also reported to be detrimental for cell nuclei health and can increase cell mortality [64]. Also, cells in solitude show less proliferation capacity and are susceptible to mortality [65], [66]. To account for these observations, the input of cell density is fuzzified using three membership functions as depicted in see Fig. 2. The parameters of ${\text{c}}_{\mathrm{clt1}}$, ${\text{c}}_{\mathrm{clt2}}$, ${\text{c}}_{\mathrm{cht1}}$, and ${\text{c}}_{\mathrm{cht2}}$ mark the boundaries of different memberships.

#### 2.2.2 Fuzzy inference, defuzzification, and cellular events

Once the cellular inputs are converted into linguistic variables, the rules given in Table 1 are used to determine the intensity of cellular actions. A given set of inputs can simultaneously trigger multiple rules. Thus, we use the weighted fuzzy mean technique to calculate the final output (see Fig. 2) [36]. The outputs of the FL controller are continuous crisp values between 0 and 1. These values are post processed to determine cellular events.

**Table 1:** Fuzzy logic rules describing the cellular reactions in response to stimulatory signals. To be concise, the combination of different inputs that results in the same cellular output is coded in certain colors; purple (~): any choice of one or more from the given inputs; blue (≈): any choice of two or more from the given inputs. The symbol (−) indicates any of the linguistic levels defined for that variable. If the rule applies for all except a certain level, it is described as ‘Not’ followed by the linguistic level, e.g. ‘Not stimulatory’ stands for all levels except stimulatory.

The proliferation, mortality, and migration are simulated as a stochastic process where the chance of occurrence at each time step is calculated,

(1) $$Proliferation\;chance=\Omega \;∙\;\left(\alpha_{P}f_{P}\right)\;∙\;\gamma_{P0}$$

(2) $$Mortality\;chance=\;\left(1+\alpha_{PM}\delta_{P}\right)\;∙\;\left(\alpha_{M}f_{M}\right)\;∙\;\gamma_{M0}$$

(3) $$Migration\;chance=\;f_{\mathrm{Mi}}$$

where $\gamma_{P0}$ and $\gamma_{M0}$ are the base chances of proliferation and mortality, respectively; $f_{P}$, $f_{M}$*,* $f_{\mathrm{Mi}}$ are the action rates calculated by the FL controller for proliferation, mortality, and migration, respectively; $\alpha_{P}$and $\alpha_{M}$ are the scale factors to scale up the controller’s outputs; Ω is a bias function; and $\delta_{P}$ and $\alpha_{PM}$ simulate the mitotic damage and its weight on the mortality chance. It is shown that shortly after proliferation, one of the daughter cells is prone to undergo apoptosis possibly due to asymmetric distribution of pro- and anti-apoptotic proteins during the final stage of cell division [67]. We account for this observation by assigning $\delta_{P}$ ($\delta_{P}=1$ if mitosis occurs, and $\delta_{P}=0$ otherwise) to one of the daughter cells after the cell cycle. Accounting for the fact that cells need a period of time for growth before the actual process of the division, we use a logistic-based bias function (Ω) to shift the probability distribution toward the end of the cell cycle (see Fig. S1-B in the supplements). The chosen logistic growth rate constrains the proliferation probability around the average time period assigned for proliferation but also leaves a degree of stochasticity in the system. Once a cell commits to proliferation, a daughter cell is created and positioned adjacent to the mother cell. Migration in the present model occurs due to contact inhibition with the chance calculated in Eq. (3). The choice of destination can be arbitrary as long as an adjacent grid is vacant. The motile cell can move one patch at a time step. If all neighboring grids are occupied, no relocation will take place.

Differentiation is simulated as a continuous process with the rate,

(4) $$Differentiation\;rate=\;{(\alpha }_{D}f_{D})\;∙\;r_{D0}$$

where $r_{D0}$ is the base rate of differentiation, $f_{D}$ is the FL controller’s output for differentiation, and α_D_ is the scale factor. Whether the maturity is below or above the maturity threshold (M_t_), $f_{D}$ can indicate the early or late differentiation rate, respectively, produced by the controller. In the present model, cell differentiation and proliferation can occur simultaneously [17].

### 2.3 Sensitivity analysis and the calibration process

The current model contains 20 free parameters (see Table S2 in the supplementary information). The empirical data to determine the values of these parameters are limited as they are either difficult to measure or represent a combination of several processes. Instead, we use a range of possible values based on empirical observations or estimations and then use the calibration process to precise their values. The empirical data for the calibration process is obtained from three sets of cell culture studies (summarized in section S4 in the supplementary information). Study 1 examines the effect of five different Mg^2+^ ions concentrations on cell population by measuring two parameters of live cell count and viability [68]. Study 2 focuses on the osteogenic differentiation process by measuring the expression of the differentiation-related markers of alkaline phosphate (ALP) and osteocalcin (OC) and growth factors at three time points of 7, 14, and 21 days as a response to two different concentrations of Mg^2+^ ions [43]. Study 3 reports live cell count at three time points of 3, 6, and 9 days for four different Mg^2+^ ions concentrations [69], [70]. The combined data provided 72 experimental measurements. All experiments were conducted with human umbilical cord perivascular (HUCPV) cells. We conduct the calibration process on the dataset of each study alone, encoded as C1, C2, and C3, as well as on the combined data of all experiments, encoded as C1-3.

**Fig. 3:**
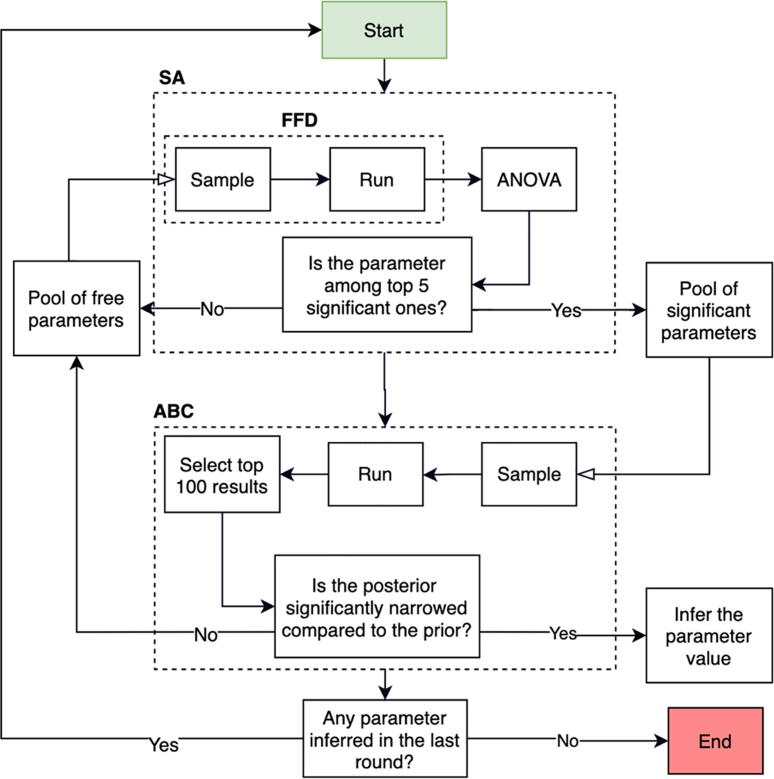
The iterative calibration process used for the parameter estimation in this study. At each iteration, the top five significant parameters are determined by the sensitivity analysis (SA) that consists of the fractional factorial design (FFD) and the analysis of variance (ANOVA). The significant parameters are sent to ABC for the parameter inference. At each iteration, the model is executed 5000 times with the parameter sets sampled from the pool of significant parameters. The posteriors are generated using the top 100 results. If the posterior is significantly narrower than the prior, the median of the posterior is accepted as the estimated value. If not, the parameter is added to the pool of the free parameters for the next round of calibration.

The approximate Bayesian calculation (ABC) is employed for parameter inference [71] (see section S7 in the supplement). However, due to the curse of dimensionality, sufficient sampling in a 20-dimensional space requires a very large number of runs, i.e. in the order of several million [71], which is impractical considering the size of the current model. To overcome this, we employ an iterative calibration process depicted in Fig. 3 that follows three main steps of (1) determining the five most important parameters using the sensitivity analysis, (2) estimating the values of the chosen parameters using ABC, and (3) updating the model with the inferred values and repeating step 1 and 2. We use fractional factorial design and analysis of variance for the sensitivity analysis (see section S6 in the supplement). The iterative calibration process ends once no new parameter is inferred at the previous iteration.

## 3 Results

In this section, we first present the results of the sensitivity analysis and the calibration process. Then, we show the improvements made on the results during iterative calibration process. Lastly, we compare the results of the simulations to the empirical data.

### 3.1 The results of the sensitivity analysis and the calibration process

**Fig. 4:**
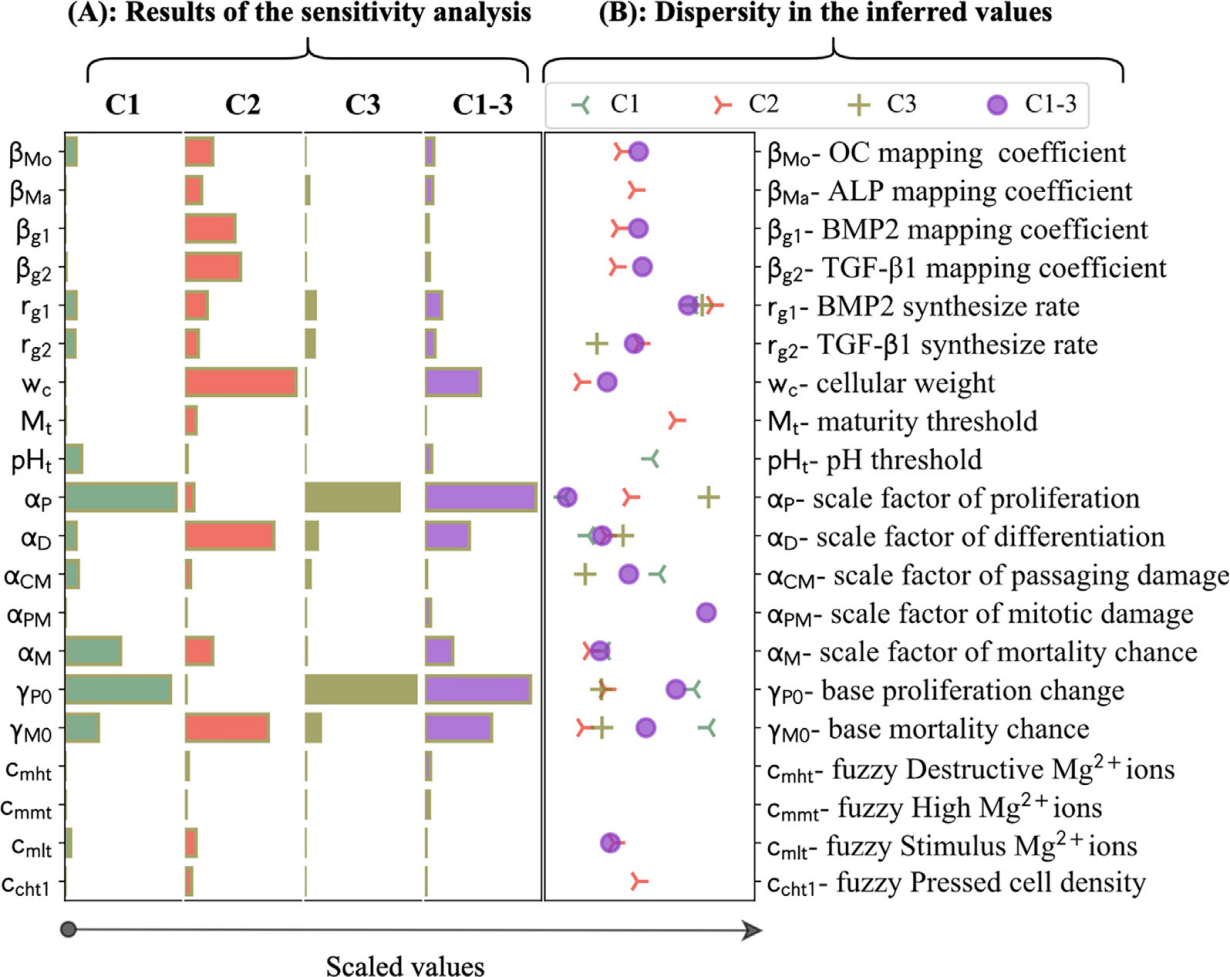
(A) The results of the sensitivity analysis, obtained during the first iteration of the calibration process for different calibration scenarios of C1, C2, C3, and C1-3. The bars indicate the significance of parameters in comparison with one another. The quantities were scaled with respect to the maximum values. (B) The comparative representation of the values estimated for the free parameters during different calibration scenarios. The values were scaled by dividing by the mean of the priors.

The complete results of the iterative calibration process are given in Fig. S2 in the supplements. It took 5, 8, 5, and 8 iterations for C1, C2, C3, and C1-3, respectively, to complete the calibration process. The significance of the parameters with respect to one another was obtained during the sensitivity analysis (see Fig. 4A). The base proliferation chance ($\gamma_{P0}$), the scale factor of proliferation ($\alpha_{P}$), and the base mortality chance ($\gamma_{M0}$) had the highest impact for C1 and C3. For C2, the top three impactful parameters were the cellular weight ($w_{c}$), the scale factor of mortality ($\alpha_{D}$), and the base mortality chance. For C1-3, the top 5 significant parameters were the combination of those in C1, C2, and C3, i.e. $\gamma_{P0}$, $\alpha_{P}$, $\gamma_{M0}$, $w_{c}$, and $\alpha_{D}$ (see Fig. 4A).

The iterative calibration process resulted in the estimation of 8 out of 20 free parameters for C1, 15 for C2, 7 for C3, and 15 for C1-3. The estimated values obtained from different calibration scenarios are given in a normalized format in Fig. 4B. The real values are presented in Table S2 in the supplement. No values were inferred for the parameters of $c_{\mathrm{mmt}}$ and $c_{\mathrm{mht}}$, and several parameters were only inferred in certain calibration scenarios. There was a large variation among the estimated values of several parameters during different calibration scenarios. Among them were $\alpha_{P}$ and $\gamma_{P0}$, connected to proliferation process, $\alpha_{\mathrm{CM}}$ and $\gamma_{M0}$, associated with mortality, and $\alpha_{D}$, related to differentiation process (see Fig. 4B).

### 3.2 The improvements on the goodness of fit (R^2^) during the iterative calibration process

**Fig. 5:**
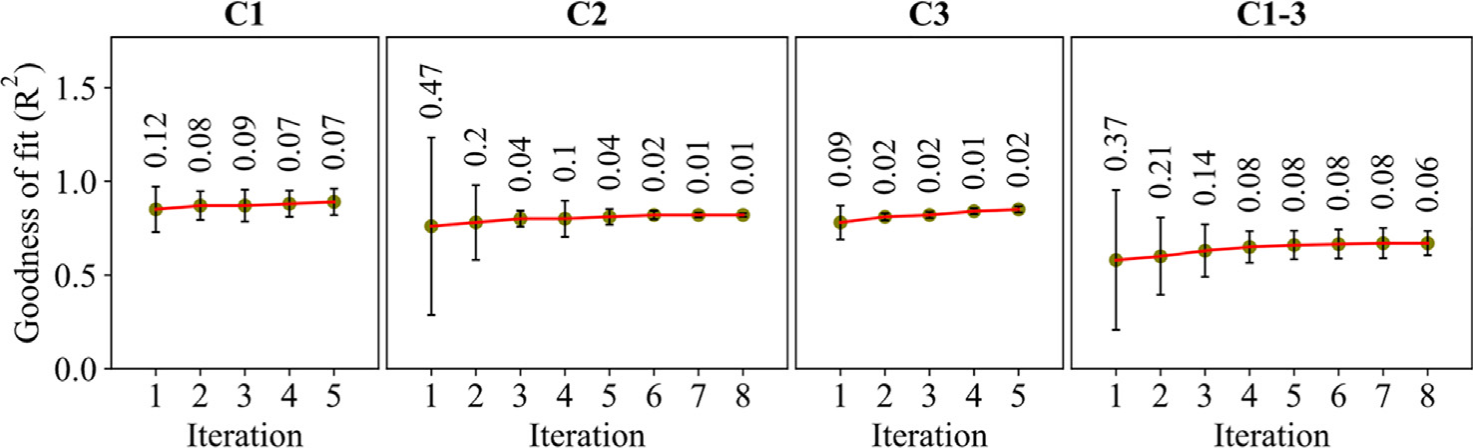
The evolution of the goodness of fit during the iterative calibration process for different calibration scenarios. The means and standard deviations were obtained from the 100 best results. The numbers above the error bars show the standard deviations.

During the iterative calibration process, the obtained values of R^2^, calculated as the normalized absolute difference between simulation results and the empirical data, were improved as depicted in Fig. 5. The standard deviation indicates the extent of the variations in the mean caused by the uncalibrated parameters. The mean and standard deviation of R^2^ respectively increased and decreased 4% and 5% for C1, 6% and 46% for study 2, 7% and 29% for C3, and 9% and 31% for C1-3 during the iteration process. For C2 and C1-3 with higher calibration iterations, the improvements made in the first five iterations accounted for 83% and 93% of the total improvements on the mean and standard deviation, respectively, during C2 and 89% and 96%, respectively, during C1-3.

### 3.3 Comparison of experimental and simulation results

**Fig. 6:**
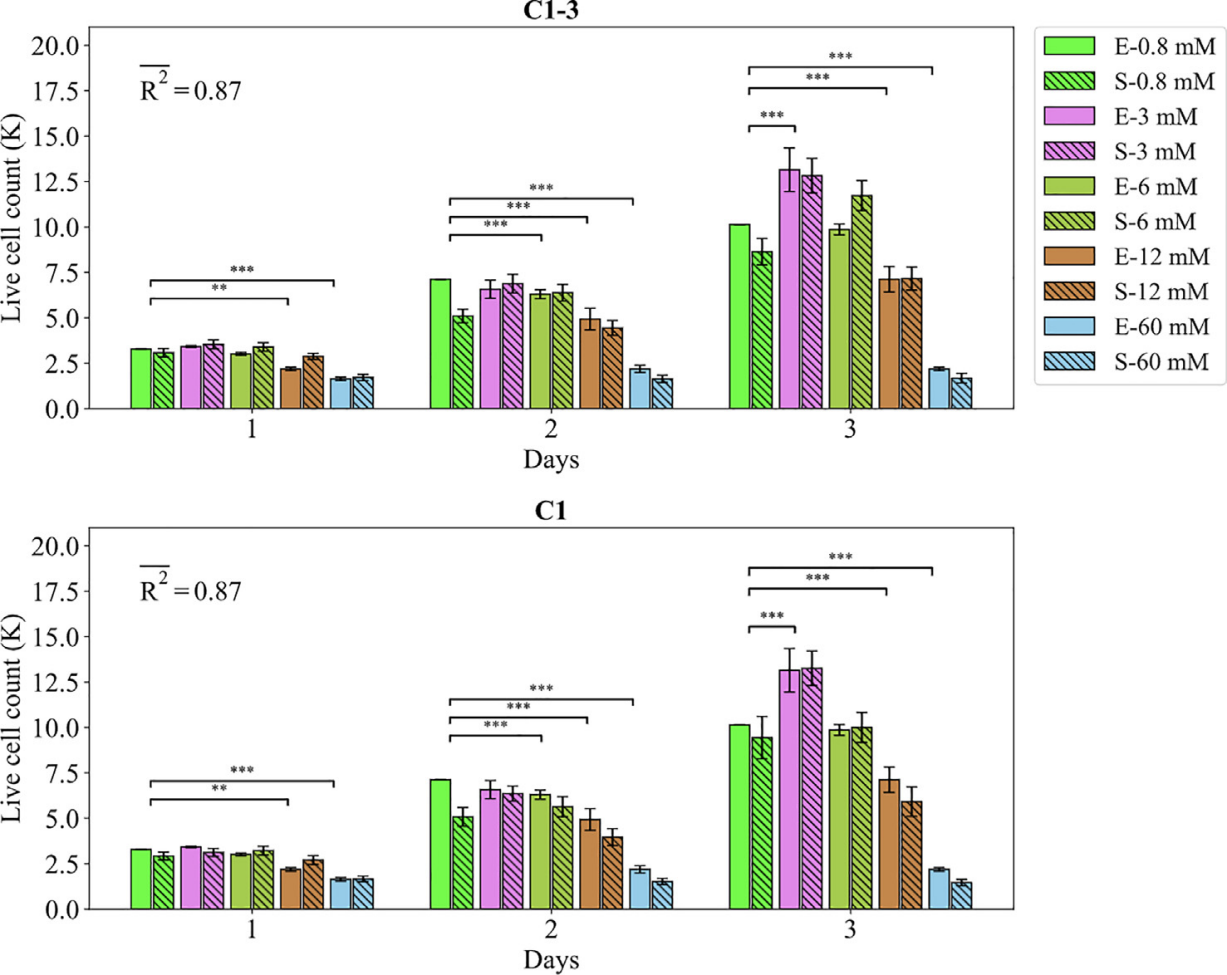
Fits of the model calibrated by C1-3 and C1 to the empirical data of study 1. Bars indicate the average of the best 100 simulations (S-) and the corresponding empirical data (E-) for increasing Mg^2+^ ion concentrations. The error bars on the empirical data shows the standard deviations. The error bars on the simulation results indicate the standard deviation of the 100 best fits. Stars indicate the statistically significant differences between values given for the empirical data compared to the control (Mg^2+^ concentration of 0.8 mM) (p < 0.05 = *; p < 0.01 = **; p < 0.001 = ***). $\overset{-}{R^{2}}$ is the average $R^{2}$ calculated for each measurement item.

**Fig. 7:**
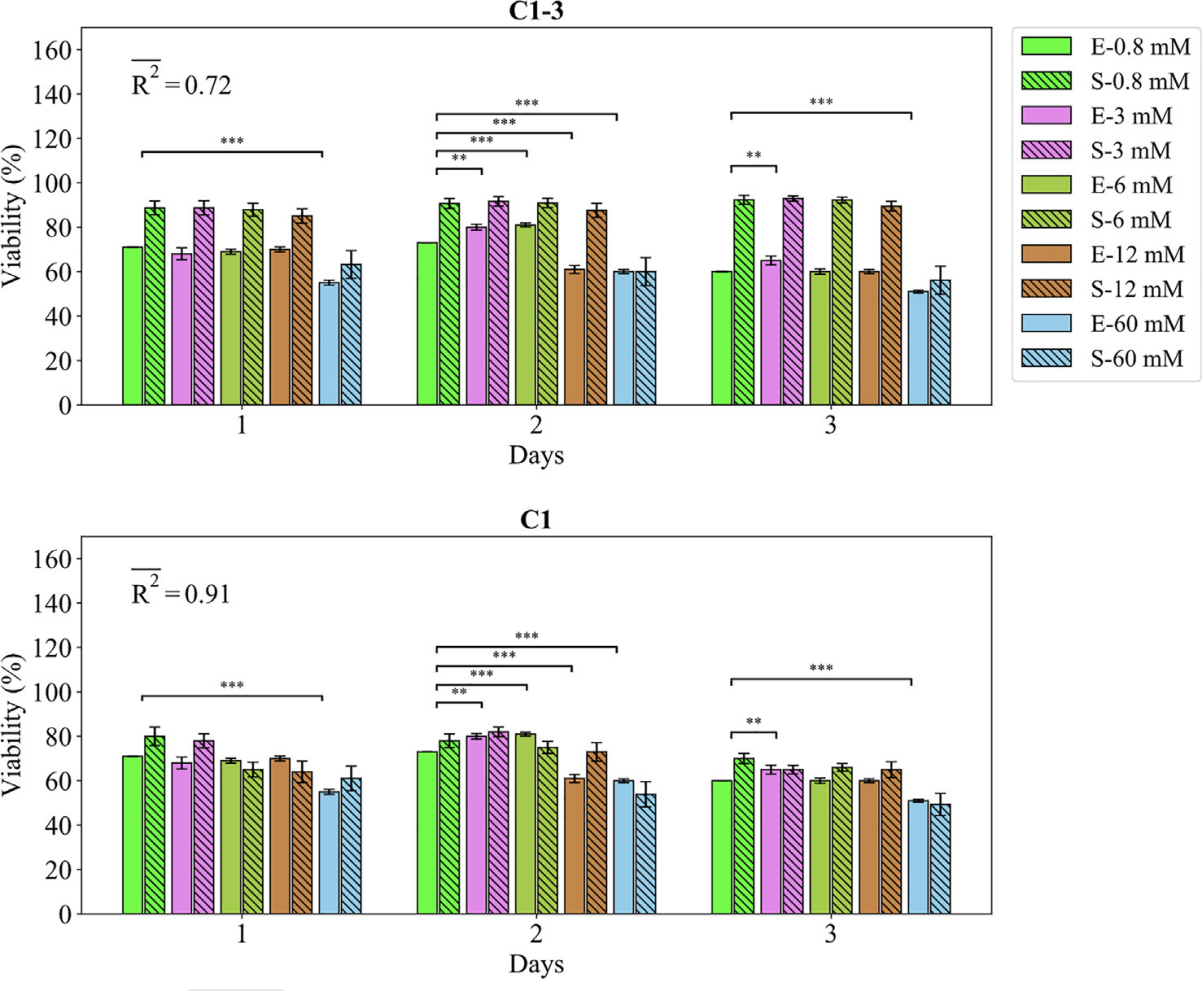
Fits of the model calibrated by C1-3 and C1 to the empirical data of study 1. Bars indicate the average of the best 100 simulations (S-) and the corresponding empirical data (E-) for increasing Mg^2+^ ion concentrations. The error bars on the empirical data shows the standard deviations. The error bars on the simulation results indicate the standard deviation of the 100 best fits. Stars indicate the statistically significant differences between values given for the empirical data compared to the control (Mg^2+^ concentration of 0.8 mM) ﻿(p < 0.05 = *; p < 0.01 = **; p < 0.001 = ***). $\overset{-}{R^{2}}$ is the average $R^{2}$ calculated for each measurement item.

**Fig. 8:**
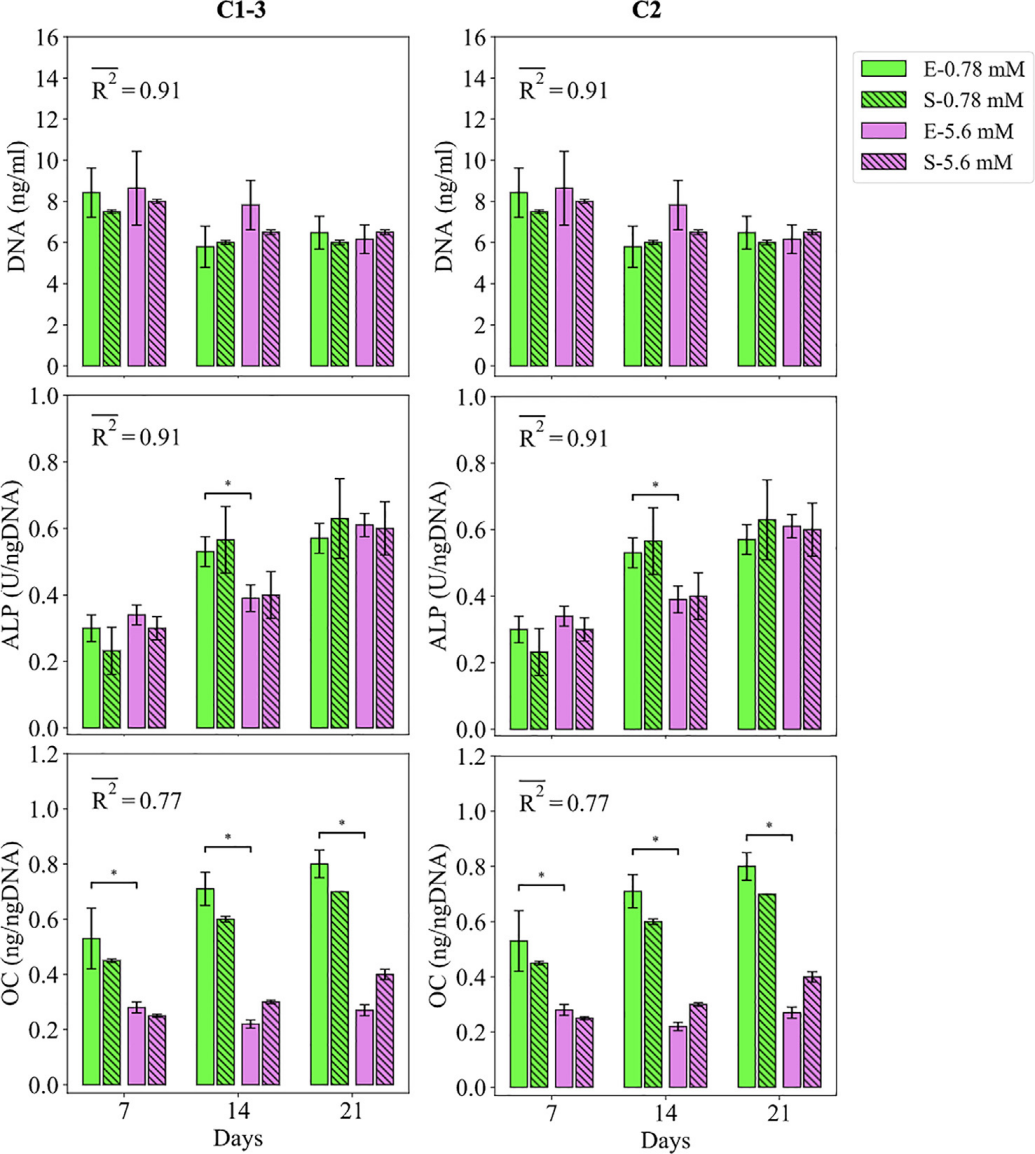
Fits of the model calibrated by C1-3 and C2 to the empirical data of study 2. Bars indicate the average of the best 100 simulations (S-) and the corresponding empirical data (E-) for different Mg concentrations. The error bars on the empirical data shows the standard deviations. The error bars on the simulation results indicate the standard deviation of the 100 best fits. Stars indicate the statistically significant differences between values given for the empirical data compared to the control (Mg^2+^ concentration of 0.78 mM) ﻿(p < 0.05 = *; p < 0.01 = **; p < 0.001 = ***). $\overset{-}{R^{2}}$ is the average $R^{2}$ calculated for each measurement item.

**Fig. 9:**
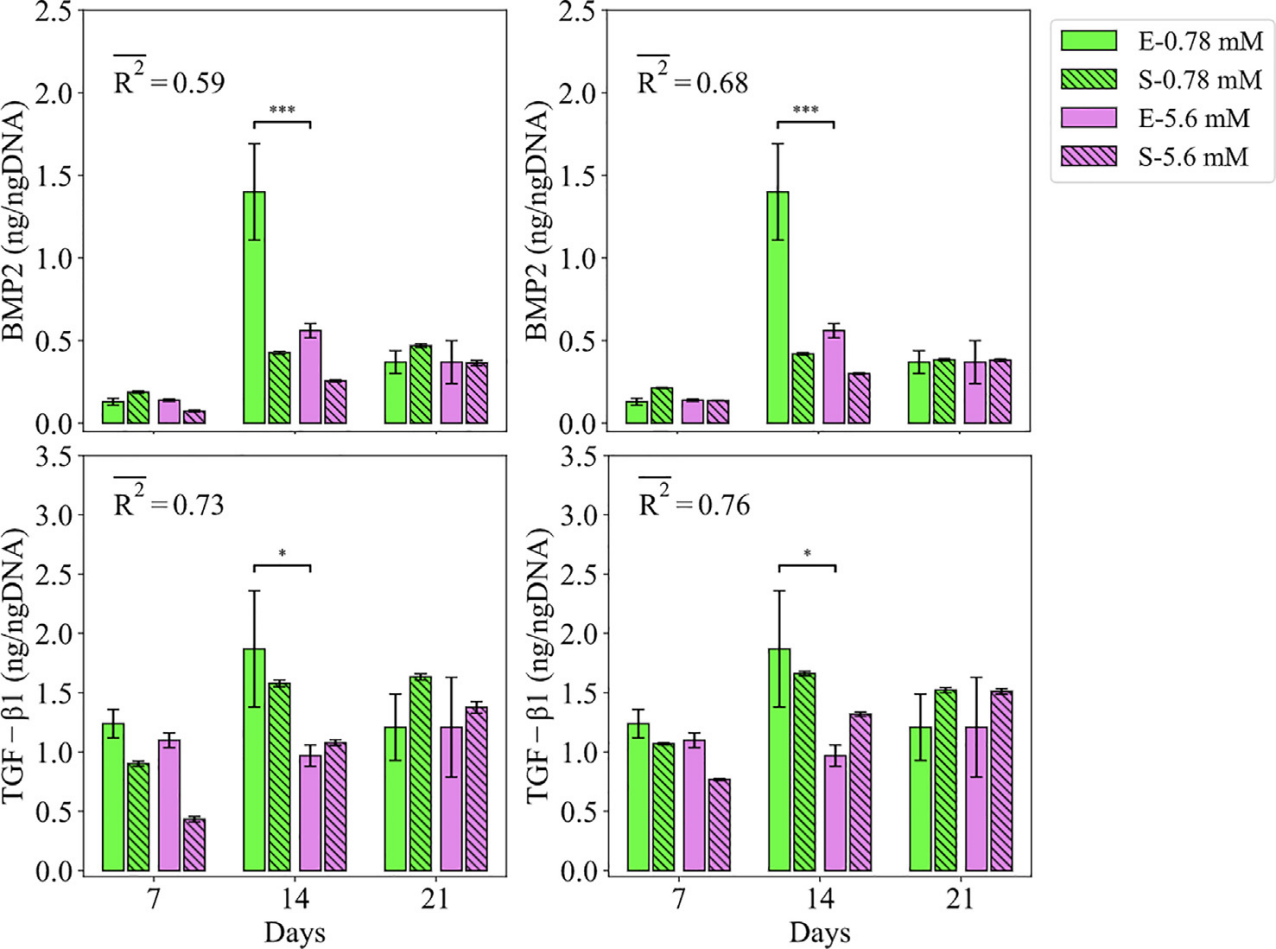
Fits of the model calibrated by C1-3 and C2 to the empirical data of study 2. Bars indicate the average of the best 100 simulations (S-) and the corresponding empirical data (E-) for different Mg concentrations. The error bars on the empirical data shows the standard deviations. The error bars on the simulation results indicate the standard deviation of the 100 best fits. Stars indicate the statistically significant differences between values given for the empirical data compared to the control (Mg^2+^ concentration of 0.78 mM) ﻿(p < 0.05 = *; p < 0.01 = **; p < 0.001 = ***). $\overset{-}{R^{2}}$ is the average $R^{2}$ calculated for each measurement item.

**Fig. 10:**
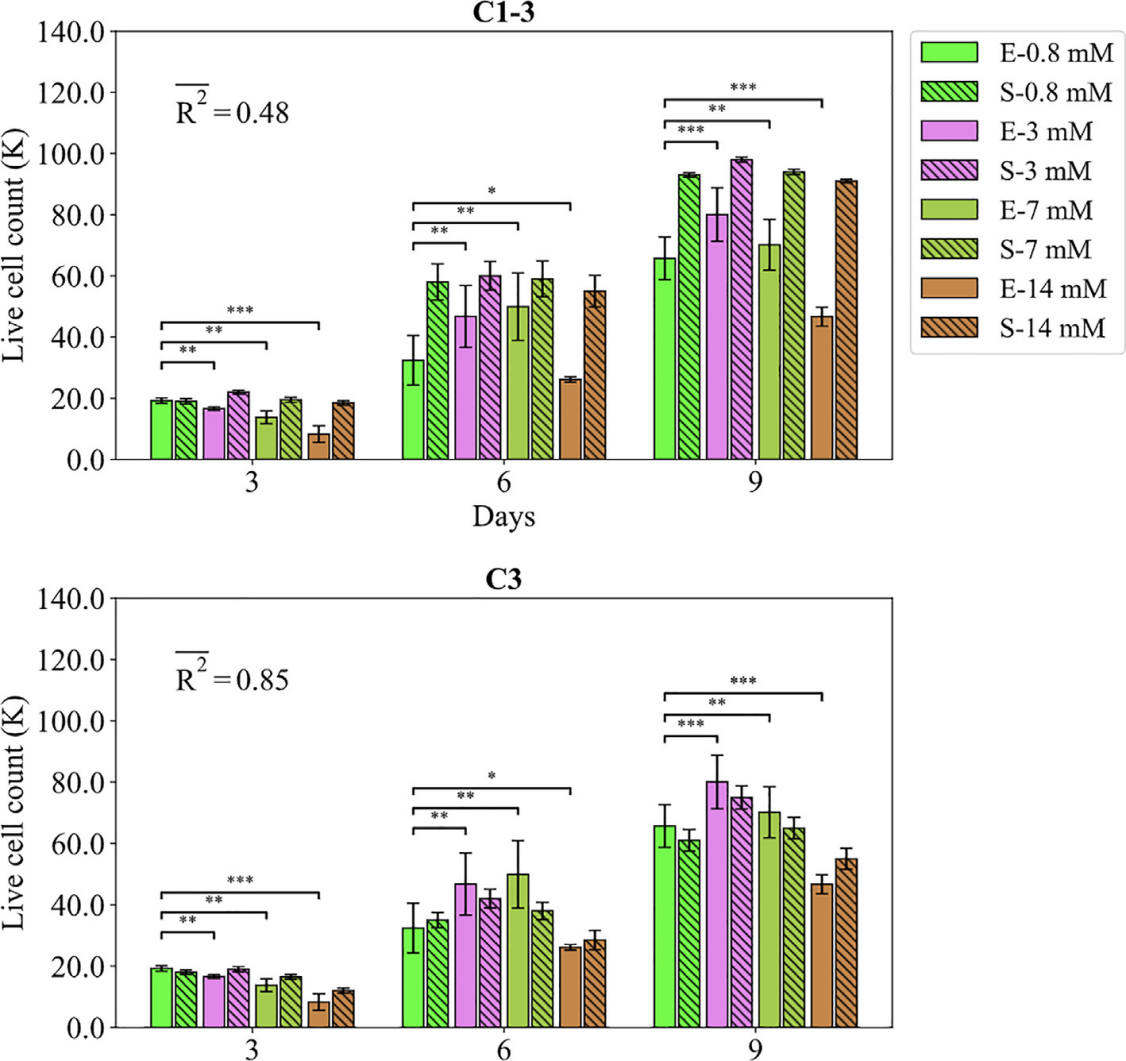
Fits of the model calibrated by C1-3 and C3 to the empirical data of study 3. Bars indicate the average of the best 100 simulations (S-) and the corresponding empirical data (E-) for different Mg concentrations. The error bars on the empirical data shows the standard deviations. The error bars on the simulation results indicate the standard deviation of the 100 best fits. Stars indicate the statistically significant differences between values given for the empirical data compared to the control (Mg^2+^ concentration of 0.8 mM) ﻿(p < 0.05 = *; p < 0.01 = **; p < 0.001 = ***). $\overset{-}{R^{2}}$ is the average $R^{2}$ calculated for each measurement item.

The agent-based model parametrized with the values accepted by ABC were compared against the empirical data as demonstrated in Fig. 6, Fig. 7, Fig. 8, Fig. 9, Fig. 10. The fits of the model to the data of each study are given in separate sections in the following.

#### 3.3.1 Study 1

The fits of the model to the data of study 1 are given in Figs. 6 and 7. The model calibrated by C1-3 produced the $R^{2}$ of 0.87 and 0.72 for the live cell count and viability, respectively. Overall, Mg^2+^ ions at the concentration of 3 mM resulted in the highest cell population followed by 6 mM, 0.8 mM (control), 12 mM, and 60 mM, which was correctly reproduced by the model (see Fig. 6). For the case of viability, the model closely reproduced the culture data given for Mg^2+^ ions concentration of 60 mM but overestimated the rest (see Fig. 7). Once calibrated against C1, the model’s predictions for the viability were considerably improved, i.e. $R^{2}$ increased from 0.72 to 0.91 (Fig. 7). The model was able to closely match the culture data for all Mg^2+^ ions. However, the simulation outcomes showed little change in the case of the live cell count comparing C1 to C1-3 (see Fig. 6).

#### 3.3.2 Study 2

The fits of the model to the data of study 2 are given in Figs. 8 and 9. The model calibrated by C1-3 resulted in an average $R^{2}$ of 0.72 for DNA content (see Fig. 8). The model was able to capture the decreasing trend of the DNA content in the course of experiments from day 7 to 21. The model was also in agreement with the culture data in terms of predicting higher DNA contents for the Mg^2+^ ions concentration of 5 mM compared to the control. However, there was an overall overestimation in the predictions made on the day 7 and 14 for both cases. Once calibrated by C2, the $R^{2}$ improved from 0.72 to 0.91, and the model’s predictions closely matched the culture data in terms of trends and exact values (see Fig. 8).

For the case of ALP and OC, the model calibrated by C1-3 resulted in an average R^2^ of 0.91, and 0.77, respectively (see Fig. 8). The culture experiments reported higher ALP for the Mg^2+^ ions concentration of 5 mM on day 7 and lower on day 14 compared to the control, which was correctly captured by the model. The OC content was reported lower for the Mg^2+^ ions concentration of 5 mM compared to the control in all three time points, which was also captured by the model (see Fig. 8). However, the model predicted an increase in OC content from day 7 to 21 which was not in close agreement with the data. Also, the model underestimated the OC content reported for the control in all three measurement points. Once calibrated by C2, the predictions of the model for the OC content were closer to the experimental data with 8% improvements in the R^2^ (see Fig. 8).

The model calibrated by C1-3 produced the average R^2^ of 0.73 and 0.59 for the growth factors of TGF-β1 and BMP2, respectively (see Fig. 9). For both TGF-β1 and BMP2, the cell culture data reported lower values for the condition of 5 mM Mg^2+^ ions compared to the control in all three time points, which was also captured by the model. However, the non-linearity shown in the data, in particular the sharp jump on day 14 of BMP2, was not seen in the model. Once calibrated by C2, the obtained average R^2^ increased from 0.73 to 0.76 for TGF-β1 and from 0.59 to 0.68 for BMP2. However, the model was still not in a close match with the culture data.

#### 3.3.3 Study 3

The fits of the model to the data of study 3 are given in Fig. 10. The model calibrated by C1-3 correctly reproduced the trend observed in the cell culture; the live cell count experienced a continuous increase from day 3 to day 9 for all Mg^2+^ ions concentrations; and the highest and lowest live cell count is obtained for 3 mM and 14 mM, respectively. The model disagreed with the data in two aspects; firstly, there was a general overestimation in the predictions of the model especially for the case of the Mg^2+^ ions concentration of 14 mM; and secondly, the culture data reported large variations across different Mg^2+^ ions concentrations, in particular on day 6 and 9, while the model’s predictions for different Mg^2+^ ions were close to one another. Once calibrated by C3, there was a substantial increase in the R^2^, i.e. from 0.48 to 0.85 (see Fig. 10). The results of the predictions were in close agreement with the culture data both in terms of trend and the exact values.

## 4 Discussion

The present computer model was initially calibrated using the accumulated data of all three experiments. The model was capable of successfully reproducing several empirical observations, more notably, the live cell count reported in study 1 and the differentiation-related markers of ALP and OC. The results of the simulation, consistent with the experiments, showed that Mg^2+^ ions within the range of 3–6 mM produce the largest hMSC population (see Fig. 6, Fig. 7, Fig. 10). Also, the model correctly reproduced the culture data in showing that while Mg^2+^ ions stimulate early differentiation, it inhibits the differentiation in the later phase (see Fig. 8). However, there was an overall discrepancy between the model’s predictions and the data for the case of viability, the DNA content, the live cell count reported in study 3, and the growth factors. To investigate whether such disagreement originated from the inherent inability of the model in capturing the complexity of the experiments or from a possible discrepancy among the given empirical data, we conducted the second round of calibration in which the model was tuned against the culture data given for each model separately (C1, C2, and C3).

The results of C1, C2, and C3 showed a significant improvement in the model’s accuracy in explaining the population-related data of DNA content, live cell count, and viability data compared to C1-3 (see section 3.3). To better understand the underlying differences between the models calibrated by different sets of data, their estimated parameter values were plotted against one another (see Fig. 4B). The observed variation in the estimated values primarily originates from the exploration of ABC in finding the global minimum based on the summary statistics [72]. However, there were meaningful patterns associated with certain parameters. It was shown that the base proliferation rate ($\gamma_{P0}$) was estimated similarly between C1 and C1-3, whereas C2 and C3 produced a notably smaller value. A similar pattern was also verified between the predictions of the models in terms of the live cell count and DNA content; the results of the live cell count predicted for study 1 were similar between C1 and C1-3 (see Fig. 6), while the results of the DNA content predicted for study 2 and live-cell count predicted for study 3 were overall higher for C1-3 compared to C2 and C3 (see Figs. 8 and 10). This suggests that the cells experimented in studies 2 and 3 were less proliferative compared to study 1. Considering that all experiments used a similar cell type (HUCPV) within the passage numbers 3 and 5, such discrepancy might step from the differences in the cell donors [73].

Another proliferation-associated parameter whose value showed a high variation across different calibration schemes was $\alpha_{P}$ (see Fig. 4B). This parameter simulates the model’s sensitivity to the stimuli related to the proliferation process including Mg^2+^ ions as given in Eq. (1). Both C2 and C3 estimated a higher value for $\alpha_{P}$ compared to C1-3 and C1 (see Fig. 4B). Simultaneously, the model calibrated by C2 and C3 produced a higher contrast across different Mg concentrations in terms of live cell count and DNA contents (see Fig. 10). Hence, it can be understood that the cells cultured in study 1 were fundamentally more sensitive to Mg^2+^ ions compared to study 1 and 2. Such an observation can stem from cell donor dependency or the aging of Mg extract due to long-term storage before cell culture, which subsequently results in less bioactive Mg^2+^ ions.

Studies 2 and 3 lacked the quantitative measurements of cell viability (see Table S1 in the supplements). Instead, a minimum threshold of 50% was used according to similar experiments [39], [40], [13], [74], in order to prevent the calibration process from producing an overall high fitness value at the cost of unrealistic cellular mortality. To satisfy this condition, C1-3 failed to closely reproduce the viability values given in study 1 (see Fig. 7). This implies that no parameter set could simultaneously satisfy the minimum viability assumed for studies 2 and 3 and the measured value in study 1. It can be seen that the cells in study 1 had a higher mortality rate than those in studies 2 and 3 (see Fig. 4B). Considering that the duration of study 1 was shorter than other studies, a higher mortality rate in the early days of culture compared to the later days can justify the observed differences. In our formulations, the factor of the cell passaging damage is assumed to cause permanent DNA damage and thereby contribute to early cellular mortality. However, assigning a large weighting factor for this process (α_CM_) results in a sudden diminishing of live cells and leaving the remaining cells in solitude. Cells in isolation experience a low proliferation and high mortality rate, according to the assumptions of the FL controller, which further contributes to the shrinkage in the cell population. Therefore, the results of our simulation suggest that either the cells cultured in the different studies had fundamental differences in their mortality behavior, or there is another factor that gradually contributes to cellular mortality in the early days of cell culture, which is not included in our formulations.

The experimental cell culture data shows that the content of the growth factors increases from day 7 to 14 and decreases from day 14 to 21. Given the fact that the reported contents for the growth factors were normalized against DNA, the observed jump on day 14 can indicate that cells were more productive within the first period of the experiment compared to the period after day 14. On the other hand, the cells were not fully differentiated before day 21, according to the results of the differentiation markers (see Fig. 8). This implies that the cells produced a higher amount of growth factors within their early differentiation phase compared to the later stage. Such an observation was not seen in the formulation of the present model (see Eq. (9) in the supplementary materials) which was adopted from the literature [75], [55]. Further investigations are required to elucidate the relationship between the cellular activity regarding growth factors production and the degree of osteogenic differentiation.

An iterative process was used to calibrate the model’s free parameters (see Fig. 3). In the proposed scheme, the iterative calibration process continued until no significant narrowing occurred. Overall, the performance of the model was considerably improved in the course of the iteration (see Fig. 5). In particular, the standard deviation of R^2^, which indicates the uncertainty in the predictions of the model, dropped to a negligible value at the end of the iterative process (see Fig. 5). The remaining variation can stem from the stochastic nature of the agent-based modeling. The results also showed that the first few iterations can account for a large portion of the total improvements; for C1 and C2, the first five iterations accounted for over 90 percent of the total improvements (see section 0). This implies that the proposed criteria to stop the calibration process from further iteration, i.e. the significance in narrowing the posterior with respect to the prior, are not optimal. Therefore, the calibration scheme proposed in this study (see Fig. 3) can be further improved in the future by adding the alteration in the mean and standard deviation of $R^{2}$ as another factor in controlling the iteration number.

In the present study, we used Markov decision process-based ABM to study the dynamics of cell population and osteoblastic differentiation. The architectural design of our ABM is reminiscent of the modeling paradigm used in reinforcement learning, where decision-maker agents interact with one other and with their micro-environment [76]. The choice of ABM, thus, can facilitate the possible subsequent transformation of our descriptive model into a predictive reinforcement model in the future. The employed FL-based approach as the decision-making center of the agents (cells) offers human-intelligible, discrete components with parsable rules. In contrast to neural networks-based simulations which outsource all the learning burden into one “Blackbox”-like network module [76], the FL-based approach is tractable and conforms to the actual properties of a system. Such an approach is well-suited for investigating and incorporating the experimental datasets which may not be in perfect agreement with each other as was the case in the present study. Once such a model is calibrated, it can serve as the natural basis for neural networks where the problem becomes more tractable for a learning algorithm

Among many limitations of the present computer model, here we discuss a few important ones. Firstly, in the implemented FL controller (see Table 1), the effect of different cellular inputs was combined using the principle of superposition. Due to the lack of information to correctly define the logic of interactions among the stimulatory factors, such an assumption is inevitable and is also made in similar studies [19], [75], [77]. We speculate that Mg2 + ions combined with TGF-β1 and BMP2 produce synergistic effects which need to be studies in the future. Secondly, we primarily investigated the bioregulatory effects of Mg^2+^ ions by applying the model to the empirical datasets with various concentrations of Mg^2+^ ions. The factor of alkalinity is also inclusively studied as it changes in a linear relationship with the concentration of Mg^2+^, which is also the case in the culture experiments. The factors of TGF-β1 and BMP2 were individually studied in the previous works [75], [55], [78] and therefore were not explicitly investigated in this study. Regarding the factor of cell density, we were not able to find any study that quantitatively reported the effect of this factor on the given cellular behaviors. This might step from the fact that a precise monitoring of cellular positioning in a colony for a long period of time, i.e. a few weeks, is not practical. Thirdly, we used discrete grids to create the simulation domain (on-lattice) instead of continuum space, known as the off-lattice approach [79]. The grid-based approach constrains agents’ movement to the defined grids while off-lattice simulation provides a continuum reach. However, the former offers superior performance compared to the latter and therefore was favored in our simulations due to the complexity of the model and the computationally demanding method employed for the calibration process. Lastly, substrate stiffness, as an important parameter in guiding osteogenic differentiation [80], was not included in the present study. This important parameter will be incorporated in our future models which will simulate the *in vivo* setup.

## 5 Conclusion

The fuzzy agent-based computer model presented in this study was generally able to reproduce the empirical observations reported for the MSC population and osteogenic differentiation. The model closely captured the nonlinearities in the regulatory effect of Mg^2+^ ions on multiple cellular processes such as cell proliferation, differentiation, and mortality. The model also showed fundamental differences between the cells cultured in different experiments in terms of proliferation capacity and sensitivity to environmental variables such as Mg^2+^ ions. Moreover, the iterative calibration approach proposed in this study was shown advantageous in improving the performance of the model and is thereby recommended over the single-round calibration method commonly used in the literature. In summary, this study shows the significance of numerical modeling in understanding and objectively explaining the experiments by special attention to the mechanisms underlying cellular processes.

## Declaration of Competing Interest

The authors declare that they have no known competing financial interests or personal relationships that could have appeared to influence the work reported in this paper.
