# Supplementary material for: Magnesium ions regulate mesenchymal stem cells population and osteogenic differentiation: A fuzzy agent-based modeling approach
Source: Comput Struct Biotechnol J. 2021 Jul 9;19:4110–22. doi: 10.1016/j.csbj.2021.07.005 (PMC8346546; doi:10.1016/j.csbj.2021.07.005)
Supplement: Supplementary data 1 [file mmc1.docx]

**Supplementary materials**

# Model initialization

A cube-shaped patch with a length of L is used for the discretization of the domain. L is calculated by taking into account the initial confluence (C_0_), initial cell count (N_0_), and culture surface area (A) of each experiment set,

| $L=\sqrt{\frac{C_{o}A}{N_{0}}}$ | (1) |
| --- | --- |

At the beginning of the simulation, cells are randomly assigned to the patches. To account for the crowding effect, the occupancy of a patch is limited to only one cell at a time [32]. However, non-agent objects such as proteins are permitted to share a patch. Agents are designed to interact with each other and with the patches in their Moore neighborhood, i.e. the 26 immediately adjacent positions. To save computational power, we only simulate 1 cm^2^ of the culture area. To partly account for this simplification, periodic boundary conditions are applied to the external surfaces of the domain [33]–[35]. The computer model is configured according to the given experiments in terms of the initial cell count, the surface area of the culture plates, and the initial concentrations of Mg and growth factors.

# Simulation of the growth factors

The dynamics of the growth factors are modeled similar to Ribeiro et al [68]. The concentration of BMP2 experience temporal and spatial variations due to cellular production, degradation, cellular consumption (both due to cellular events and background consumption), and diffusion,

| $\frac{\partial c_{i}}{\partial t}=-\left( \frac{{\partial c}_{i}^{events}}{\partial t}+\frac{{\partial c}_{i}^{background}}{\partial t} \right)-\frac{{\partial c}_{i}^{degradation}}{\partial t}+\frac{{\partial c}_{i}^{production}}{\partial t}+\frac{{\partial c}_{i}^{diffusion}}{\partial t} ,i\in g1,g2$ | (2) |
| --- | --- |

where g1, g2 are the abbreviations for BMP2 and TGF-β1*,* respectively. However, in our simulation that is calibrated against *in vitro* experiments, the importance of spatial variation can be neglected. Cells (as the source of the growth factors) are randomly distributed within the simulation domain with a minimum of four cells in one patch neighborhood (15 percent confluence). Considering the diffusion coefficients of 0.36 mm^2^ h^-1^ for BMP2 [69] and 0.094 mm^2^ h^-1^ for TGF-β1 (estimated using the molecular weight of 25 kDa [70]), within the one-hour time step of the simulation, the concentration of the growth factors reaches equilibrium within the simulation domain. Also, the heterogeneity among cells with respect to the rate of production and consumption is low. Therefore, we simplify Eq. (2) to ordinary differential equations by averaging over the spatial variable. Similar to [68], we assume that cellular consumption of BMP2 mainly occurs due to the differentiation process,

| $\frac{{\partial c}_{g1}^{events}}{\partial t}=\tilde{r_{d}}\left( c_{g1}-c_{g1}^{min} \right)$ | (3) |
| --- | --- |

where $c_{g1}^{min}$ is the low boundary of the physiological concentration of BMP2. $\tilde{r_{d}}$ is the average rate of cellular differentiation over all live cells. Since differentiation is simulated as a continuous process in our simulation, the semantics of the Eq. (3) differ from [68] as they assumed an ﻿indicator function to represent the sudden differentiation occurrence. However, due to the linearity of the equation, both approaches should derive similar results. In contrast to BMP2, the consumption of TGF-β1 is assumed to occur mainly due to the proliferation process,

| $\frac{{\partial c}_{g2}^{events}}{\partial t}=\tilde{\delta_{p}}\left( c_{g2}-c_{g2}^{min} \right)$ | (4) |
| --- | --- |

where $\tilde{\delta_{p}}$ is calculated as,

| $\tilde{\delta_{p}}=\frac{1}{n}\sum_{i = 1}^{n} \delta_{p}^{i}$ | (5) |
| --- | --- |

where n is the number of live cells, and $\delta_{p}^{i}$ is an indicator function representing the *i*th cell. $\delta_{p}^{i}$ is set to 1 if the *i*th cell commits proliferation and 0 otherwise [68]. $C_{g2}^{min}$ is the minimum physiological concentration of TGF-β1. The background consumption, due to ﻿reversibly binding to cell receptors, is modeled similar to Ribeiro et al [68] using ﻿Michaelis-Menton kinetics,

| $\frac{{\partial c}_{i}^{background}}{\partial t}=\frac{V_{i}^{max}c_{i}}{K_{i}^{b}+c_{i}}c_{c} ,i\in g1,g2$ | (6) |
| --- | --- |

where $V_{i}^{max}$ is the maximum rate at which the binding process occurs, $K_{i}^{b}$ is the Michaelis constant, and $c_{c}$ is the cell concentration over the simulation domain [68]. $c_{c}$ is calculated,

| $c_{c}=\frac{n_{c}}{{10}^{6}}v_{m}$ | (7) |
| --- | --- |

where $n_{c}$ is the number of cells, $v_{m}$ is the volume of the growth medium, 10^6^ and is the maximum cell number reached in 1mL of growth medium [72]. The protein degradation due to half-life is simulated [68],

| $\frac{{\partial c}_{i}^{degradation}}{\partial t}={-\frac{ln2}{T_{i}^{1/2}}c}_{i} ,i\in g1,g2$ | (8) |
| --- | --- |

where $T_{i}^{1/2}$ is the half-life of the soluble. The production of growth factors due to cell activity is simulated as [68],

| $\frac{{\partial c}_{i}^{production}}{\partial t}=r_{i}\frac{c_{i}}{K_{i}^{p} +c_{i}} c_{c} ,i\in g1,g2$ | (9) |
| --- | --- |

where $r_{i}$ is the maximum rate of production, and $K_{i}^{p}$ is the Michaelis constant. The described equation is solved at each time step of the simulation by receiving the initial density of the growth factors within the patches of the agent-based model.

# Software specification

The ABM developed in this study using the free open-source library of CppyABM [31]. The fuzzy-logic controller is implemented in FuzzyLite Library [35]. The sensitivity analysis is conducted using a free Python package developed in-house named barneySA [2]. A free Python package developed in-house named ABayesianC [5] is employed for the calibration process. The simulations run using Message Passing Interface (MPI) to manage large computational demands. Maxwell computational resources operated at Deutsches Elektronen-Synchrotron (DESY) are employed with more than 100 CPUs for our computations.

# Empirical data

In principle, the HUCPV cells of a continuous culture (passage three to five) were grown in α Minimal Essential Medium (MEM) with 15% stem cell Fetal Bovine Serum (scFBS) under cell culture conditions at 37°C, 5% CO2 and humidified atmosphere. The medium was changed every two to three days. The cells were counted by a Casy cell counter and analyzer (Omni life science, Bremen, Germany) which also allows the quantification of viable cells. In order to measure the activity of alkaline phosphatase, a photometric test kit BioAssay System (Hayward, USA) and an Alizarin Red staining were used. The quantity of OC in the supernatant of the cell culture was determined by ELISA (Human Osteocalcin Instant ELISA, eBioscience, Frankfurt, Germany). The viability reports the percentage of live cell count to the total cell count and is used to evaluate the mortality simulated in the current model. DNA content linearly correlates with the live cell count by taking into account the cell weight ($w_{c}$) [81]. ALP and OC are reported as the normalized values against DNA content [42]. Therefore, these parameters linearly correlate with maturity which is a normalized indicator of differentiation in our simulations. Two parameters of *β_Ma_* and *β_Mo_* map maturity to ALP and OC, respectively. ALP is generally considered as an early differentiation marker. Thus, we assume that ALP increases with maturity until the early differentiation threshold (M_t_) and then stays constant afterward. However, OC, as the late marker of differentiation, continuously increases with maturity in its whole range. The quantities of TGF-β1 and BMP2 are reported as the normalized values against the housekeeping genes across all experiments [42]. Therefore, we normalize the simulated quantities of TGF-β1 and BMP2 with respect to the live cell count and assume a linear relationship between these quantities and the empirically measured counterparts with the coefficients of $\beta_{t}$ and $\beta_{b}$, respectively.

Table S1: The summary of the specifications of the cell culture experiments.

| Study | Mg^2+^ ions (mM) | Cultured cells (10^3^) | Initial confluence (%) | Culture surface (cm^2^) | Measurements (day) | Measurements | Ref. |
| --- | --- | --- | --- | --- | --- | --- | --- |
| 1 | 0.80, 3.04, 6.08, 12.16, 60.80 | 10 | 20 | 3.65 | 1, 2, 3 | live cell count, viability | [78] |
| 2 | 0.80, 5.60 | 50 | 85 | 9.60 | 7, 14, 21 | DNA, ALP, OC, BMP2, TGF-β1 | [42] |
| 3 | 0.80, 3.60, 7.20, 14.40 | 5 | 15 | 1.90 | 3, 6, 9 | live cell count | [79], [80] |

# Model’s parameters

The parameters of the model are either obtained from the literature or defined as free parameters (see Table S2) and estimated during the calibration process.

Table S2: The list of the free parameters and the inferred values during different calibration schemes. Those with ‘-‘ were not inferred during that particular calibrations scenario.

| **Parameter** | **Label** | **Value range/ prior** | **Reference** | **Inferred value** | | | |
| --- | --- | --- | --- | --- | --- | --- | --- |
|  |  |  |  | C1 | C2 | C3 | C1-3 |
| $\gamma_{M0}$ | Base mortality chance | 0.0001-0.001 /hour | [9] [10] | 0.00086 | 0.0002 | 0.0003 | 0.00053 |
| $\gamma_{P0}$ | Base proliferation change | 0.021-0.083 /hour | [11] | 0.074 | 0.03 | 0.028 | 0.065 |
| $\alpha_{M}$ | Scale factor of mortality chance | 0-20 | Estimated | 5.68 | 4.24 | - | 5.26 |
| $\alpha_{P}$ | Scale factor of proliferation chance | 1-20 | Estimated | 1.98 | 8.42 | 16.4 | 2.23 |
| $\alpha_{D}$ | Scale factor of differentiation rate | 1-10 | Estimated | 2.3 | 3.2 | 4.1 | 3 |
| $\alpha_{\mathrm{CM}}$ | Scale factor of cell passaging damage | 0-100 | Estimated | 54.65 | - | 19.26 | 40 |
| $\alpha_{\mathrm{PM}}$ | Scale factor of mitotic damage | 0-10 | Estimated | - | - | - | 7.7 |
| $\beta_{Ma}$ | ALP mapping coefficient | 0.5-1 | Estimated | - | 0.64 | - | - |
| $\beta_{Mo}$ | OC mapping coefficient | 0.5-1 | Estimated | - | 0.54 | - | 0.67 |
| $\beta_{g2}$ | TGF-β1 mapping coefficient | 0.001-0.1 | Estimated | - | 0.034 | - | 0.047 |
| $\beta_{g1}$ | BMP2 mapping coefficient | 0.001-0.1 | Estimated | - | 0.035 | - | 0.035 |
| $\mathrm{pH}_{t}$ | pH threshold | 8.5-9.5 | [10][12] | 9.21 | - | - | - |
| $M_{t}$ | Maturity threshold | 0.5-1 | Estimated | - | 0.93 | - | - |
| $c_{cht1}$ | Fuzzy *Pressed* cell density | 0.7-1 | Estimated | - | 0.75 | - | - |
| $c_{mlt}$ | Fuzzy *Stimulus* Mg^2+^ ions | 2-10 mM | [7] | - | 3.95 | - | 3.75 |
| $c_{mmt}$ | Fuzzy *High* Mg^2+^ ions | 10-20 mM | [7] | - | - | - | - |
| $c_{mht}$ | Fuzzy *Destructive* Mg^2+^ ions | 20-40 mM | [8] | - | - | - | - |
| $w_{c}$ | Cellular weight | 0.01-1 ng | [16] | - | 0.17 | - | 0.3 |
| $r_{g1}$ | BMP2 synthesize rate | 0.1-0.5 ng/mL/normalized cell density | Estimated | 0.42 | 0.48 | 0.45 | 0.41 |
| $r_{g2}$ | TGF-β1 synthesize rate | 500-2000 ng/mL/ normalized cell density | Estimated | - | 1127 | 621 | 1065 |

## Parameters of the fuzzy controller

Several parameters are defined during the formulation of the fuzzy membership functions (see section 2.1). For the case of the cellular density, the parameter of $c_{clt1}$ is defined to mark the beginning of *favorable* membership. Similar to [64]. we assume a minimum of two neighbors for cells to survive solitude which results in $c_{clt1}$= 0.077. $c_{clt2}$ is set to 0.115 accounting for three neighbor cells which marks the start of the plateau of Favorable membership. The free parameter of $c_{cht1}$ as a free parameter within the range of 0.7-1 to mark the start of Pressed membership. For the case of $c_{cht1}$ < 1, the Pressed membership reaches its plateau by the presence of one more neighbor cell, i.e. $c_{cht2}$ = $c_{cht1}$+ 0.039. For the case of Mg^2+^ ions, the parameters of $c_{mlt}$, $c_{mmt}$, and $c_{mht}$ are defined as free parameters (see Table S1). The parameter of A_t_ that denotes the threshold alkalinity is estimated 0.11 accounting for the alkalinity caused by Mg^2+^ ions at the concentration of 8.5 mM (see section 2.1 and Figure S1-A). The estimation is done by considering that the maximum pH can reach 9.5 [12].

## Parameters of the cellular events

The parameters defined in section 2.2 are elaborated here. The base rate of proliferation ($\gamma_{P0}$), as a free parameter, is set to the range of 0.021-0.083 /hour which corresponds to the mitosis time of 24-72 hours [68]. The base rate of mortality ($\gamma_{M0}$) is set to 0.0001-0.001 /hour which is estimated by considering 50-100% for viability [69] [40]. The scale factors of $\alpha_{P}$, $\alpha_{D}$, and $\alpha_{M}$ that scale the fuzzy logic controller’s outputs for proliferation, differentiation, and mortality, respectively, are defined as free parameters (see Table S2). The base rate of differentiation ($r_{D0}$) is set to 0.0014/hour considering that the full differentiation process takes one month [30]. The base chance of DNA damage due to passaging is defined as $\gamma_{c}=\alpha_{CM}\gamma_{M0}$ where $\alpha_{CM}$ is defined as a free parameter. The rate of recovery from alkaline conditions (r_r_) is set to 0.003 /hour accounting for the fact that cells have recovered from alkalinity caused by Mg^2+^ ions concentration of 25 mM in the span of three weeks [9].

## Parameters of the growth factor model

The parameters defined in section S‎2 are elaborated here. The minimum physiological concentration of BMP2 ($c_{g1}^{min}$) and TGF-β1 ($c_{g2}^{min}$) is set to 0.008 ng/mL [68] and 14.2 ng/mL [71], respectively. The values of $V_{i}^{max}$ and $K_{i}^{b}$ are adopted from [68] assuming similar consumption rates for both growth factors. The half-life ($T_{i}^{1/2}$) of the BMP2 and TGF-β1 is set to 10.08 hours [68] and 10 mins [73], respectively. $K_{i}^{p}$ is chosen in such a way that the saturation concentration occurs in the physiological range of 14.2-36.3 ng/mL for TGF-β1 [71] and 0.008-0.5 ng/ml for BMP2 [53], [74]. The base rate of BMP2 and TGF-β1 synthesize (r_g1_ and r_g2_, respectively) are estimated to keep the concentration within the physiological range for the scenario with no stimulus of Mg^2+^ ions, i.e. control experiment (see Table S2).

## Parameters defined to compare the simulations results with the empirical data

The parameters defined in section ‎S4 are elaborated here. The cell weight ($w_{c}$), which is used to map live cell count to DNA, is defined as a free parameter (see Table S2). *β_Ma_* and *β_Mo_,* which are used to map maturity to ALP and OC, respectively, are also defined as free parameters (see Table S2). The measured and simulated TGF-β1 and BMP2 are linearly correlated using the free parameters of $\beta_{g1}$ and $\beta_{g2}$, respectively, (see Table S2).

# Sensitivity analysis

Fractional factorial design (FFD) aims at exposing the most important features of the system with minimum experiments [1]. We conduct a two-level FFD with the resolution of V. Our FFD setup consists of 64 runs with the replication number of two in order to partially account for the inherent stochasticity in the model. Analysis of variance is used to determine the importance of each parameter by calculating the percentage of the total sum of square [1],

| $TSS=\frac{SS_{F}}{SS_{T}}\times100$ | (10) |
| --- | --- |

where $SS_{T}$ is the total sum of squares of the deviation about the mean:

| $SS_{T}=\sum_{i=1}^{N} \left[ y_{i}-\overline{y} \right]^{2}$ | (11) |
| --- | --- |

where N is the total number of runs, $y_{i}$ is the output of the *i*th run averaged over replicas, and $\overline{y}$ is the mean over $y_{i}$.

In Eq. (10), $SS_{F}$ indicates the influence of the parameter:

| $SS_{F}=\sum_{i=1}^{L} N_{P,i}\left[ \overline{y}_{P,i}-\overline{y} \right]^{2}$ | (12) |
| --- | --- |

where $\overline{y}_{F,i}$ is the mean output obtained for each level of each parameter, L is the number of levels of each parameter which is 2 in this study, and $N_{P,i}$ is the number of runs at each level of each parameter.

# Approximate Bayesian calculation

ABC is widely used to calibrate ABMs in which the likelihood computation is practically impossible but the simulation from the model is straightforward [3], [4]. The paradigm of ABC follows three steps; sample from prior; simulate the ABM for each sample; and extract the posterior distribution by selecting the top fits. We specify uniform priors for each of the parameters with the bounds given in Table S1. Five thousand parameter values were drawn uniformly from the priors and the corresponding parameter sets were simulated twice per sample set.

Each simulation result is a vector of summary statistics ($s_{1},s_{2},\ldots,s_{m}$) that needs to be evaluated against the corresponding empirical data ($s_{e,1},s_{e,2},\ldots,s_{e,m}$). The following goodness of fit (R^2^) is used to evaluate the simulations results with reference to the observation,

| $R^{2}=1-\frac{1}{m} \sum_{i=1}^{m} \frac{{\vert s}_{i}-s_{e,i}\vert}{s_{e,i}}$ | (13) |
| --- | --- |

which prevents one measurement factor to dominate another by having $s_{e,i}$ factor in the denominator. We execute the model 5000 times at each iteration and choose the top 100 model runs that produce the best fit [79]. This method is known as the rejection algorithm which is the most fundamental ABC method in sampling from the posterior distribution (Beaumont, 2018). The obtained posterior distributions were used for the value inference. Levene’s test was used to examine whether the marginal posteriors are significantly narrower than priors [79].

# Supplementary figures


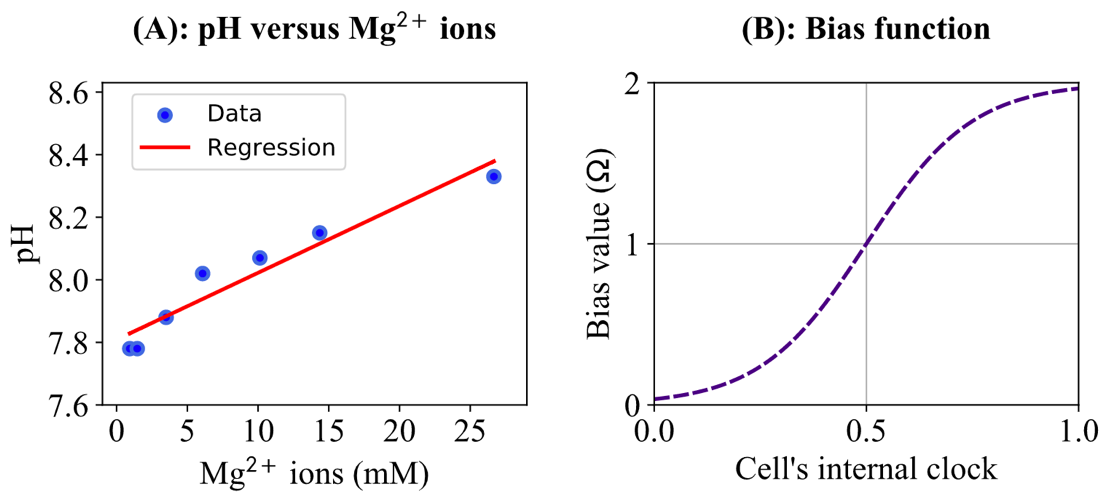


Figure S1: (A) a linear regression (slope 0.021, intercept 7.83, and R-value = 0.96) was used to map pH values to Mg concentrations according to the data derived from [6]. (B) a logistic-based growth factor with a growth rate of 8 was defined to shift the probability distribution toward the end of the proliferation cycle accounting for the fact that cells require a time span for growth before the actual division process. The internal clock resets once the cell commits to mitosis.


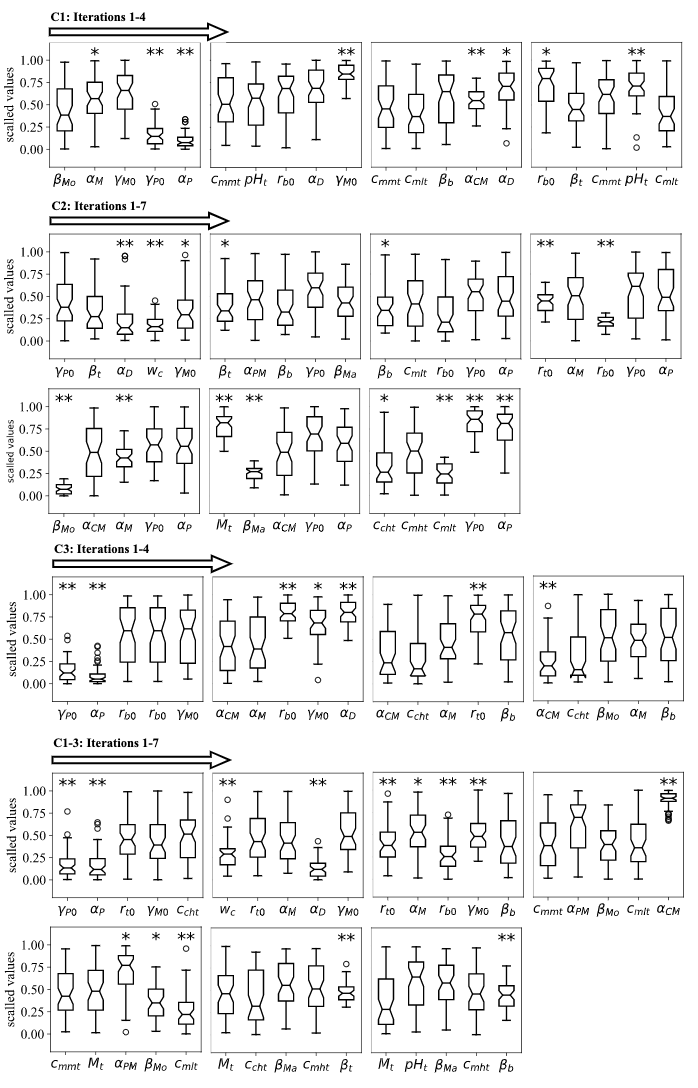


Figure S2: Posterior distributions of the parameter values estimated during different calibration scenarios of C1, C2, C3, and C1-3. The values are scaled by dividing by the range of the priors. The results of the consecutive runs are listed from left to right. The parameters that appeared in the early iterations had a higher impact on the model’s predictions. The significance in narrowing is marked by * for $p<0.01$ and ** for $p<0.0001$. Boxes show 1^st^ and 3^rd^ quartiles. Whiskers mark the limits of 1.5 × interquartile range. Hedges indicate medians.

**References**

[1] F. D. Van Voorhees and A. T. Bahill, “Sensitivity analysis by design of experiments,” pp. 58–65, 2002, doi: 10.1109/ecbs.1995.521841.

[2] J. Nourisa, “janursa/barneySA v1.0.1,” Feb. 2021, doi: 10.5281/ZENODO.4552580.

[3] E. van der Vaart, M. A. Beaumont, A. S. A. Johnston, and R. M. Sibly, “Calibration and evaluation of individual-based models using Approximate Bayesian Computation,” *Ecol. Modell.*, vol. 312, pp. 182–190, 2015, doi: 10.1016/j.ecolmodel.2015.05.020.

[4] A. P. Browning, S. W. McCue, R. N. Binny, M. J. Plank, E. T. Shah, and M. J. Simpson, “Inferring parameters for a lattice-free model of cell migration and proliferation using experimental data,” *J. Theor. Biol.*, vol. 437, pp. 251–260, 2018, doi: 10.1016/j.jtbi.2017.10.032.

[5] J. Nourisa, “janursa/ABayesianC v1.0.8,” Feb. 2021, doi: 10.5281/ZENODO.4552572.

[6] L. Wu, F. Feyerabend, A. F. Schilling, R. Willumeit-Romer, and B. J. Luthringer, “Effects of extracellular magnesium extract on the proliferation and differentiation of human osteoblasts and osteoclasts in coculture,” *Acta Biomater.*, vol. 27, no. 294--304, 2015.

[7] X. Zhang *et al.*, “Ion channel functional protein kinase TRPM7 regulates Mg ions to promote the osteoinduction of human osteoblast via PI3K pathway: In vitro simulation of the bone-repairing effect of Mg-based alloy implant,” *Acta Biomater.*, vol. 63, no. 6, pp. 369–382, 2017, doi: 10.1016/j.actbio.2017.08.051.

[8] A. Burmester, R. Willumeit-Römer, and F. Feyerabend, “Behavior of bone cells in contact with magnesium implant material,” *J. Biomed. Mater. Res. - Part B Appl. Biomater.*, vol. 105, no. 1, pp. 165–179, 2015, doi: 10.1002/jbm.b.33542.

[9] C. Yang, G. Yuan, J. Zhang, Z. Tang, X. Zhang, and K. Dai, “Effects of magnesium alloys extracts on adult human bone marrow-derived stromal cell viability and osteogenic differentiation,” *Biomed. Mater.*, vol. 5, no. 4, 2010, doi: 10.1088/1748-6041/5/4/045005.

[10] D. Maradze, D. Musson, Y. Zheng, J. Cornish, M. Lewis, and Y. Liu, “High Magnesium Corrosion Rate has an Effect on Osteoclast and Mesenchymal Stem Cell Role during Bone Remodelling,” *Sci. Rep.*, vol. 8, no. 1, pp. 1–15, 2018, doi: 10.1038/s41598-018-28476-w.

[11] D. Baksh, R. Yao, and R. S. Tuan, “Comparison of proliferative and multilineage differentiation potential of human mesenchymal stem cells derived from umbilical cord and bone marrow,” *Stem Cells*, vol. 25, no. 6, pp. 1384–1392, 2007.

[12] A. M. S. Simão, M. Bolean, M. F. Hoylaerts, J. L. Millán, and P. Ciancaglini, “Effects of pH on the Production of Phosphate and Pyrophosphate by Matrix Vesicles’ Biomimetics,” *Calcif. Tissue Int.*, vol. 93, no. 3, pp. 222–232, 2013, doi: 10.1007/s00223-013-9745-3.

[13] M. Knippenberg, M. N. Helder, B. Z. Doulabi, P. Wuisman, and J. Klein-Nulend, “Osteogenesis versus chondrogenesis by BMP-2 and BMP-7 in adipose stem cells,” *Biochem. Biophys. Res. Commun.*, vol. 342, no. 3, pp. 902–908, 2006.

[14] K. Sarahrudi *et al.*, “Elevated transforming growth factor-beta 1 (TGF-β1) levels in human fracture healing,” *Injury*, vol. 42, no. 8, pp. 833–837, 2011, doi: 10.1016/j.injury.2011.03.055.

[15] B. J. C. Luthringer and R. Willumeit-Römer, “Effects of magnesium degradation products on mesenchymal stem cell fate and osteoblastogenesis,” *Gene*, vol. 575, no. 1, pp. 9–20, 2016, doi: 10.1016/j.gene.2015.08.028.

[16] et al Lodish H, Berk A, Zipursky SL, “Molecular Cell Biology. 4th edition,” in *The Molecules of Life*, New York: W. H. Freeman, 2000.
